# Autophagy, Mesenchymal Stem Cell Differentiation, and Secretion

**DOI:** 10.3390/biomedicines9091178

**Published:** 2021-09-07

**Authors:** Mikhail Menshikov, Ekaterina Zubkova, Iuri Stafeev, Yelena Parfyonova

**Affiliations:** Laboratory of Angiogenesis, Institute of Experimental Cardiology, National Medical Research Center of Cardiology, Russian Health Ministry, 121552 Moscow, Russia; cat.zubkova@gmail.com (E.Z.); yuristafeev@mail.ru (I.S.); yeparfyon@mail.ru (Y.P.)

**Keywords:** mesenchymal stem cells, autophagy, differentiation, signal transduction, immunomodulation

## Abstract

Mesenchymal stem cells (MSC) are multipotent cells capable to differentiate into adipogenic, osteogenic, and chondrogenic directions, possessing immunomodulatory activity and a capability to stimulate angiogenesis. A scope of these features and capabilities makes MSC a significant factor of tissue homeostasis and repair. Among factors determining the fate of MSC, a prominent place belongs to autophagy, which is activated under different conditions including cell starvation, inflammation, oxidative stress, and some others. In addition to supporting cell homeostasis by elimination of protein aggregates, and non-functional and damaged proteins, autophagy is a necessary factor of change in cell phenotype on the process of cell differentiation. In present review, some mechanisms providing participation of autophagy in cell differentiation are discussed

## 1. Introduction

The problem of damaged tissue repair is always an issue of great actuality. To date, extensive experience has been accumulated in the use of progenitor cells to restore the function of damaged tissues.

Now it is revealed that mesenchymal stem cells (MSC) represent a promising direction for regenerative medicine, having a potential for repair of damaged or pathologically modified tissues [1,2]. This approach has two general possibilities of therapeutic action induced by transplanted cells: the cell differentiation (to substitute a part of damaged tissue) [3], and a paracrine action (trophic effect) in damaged area. MSC can be immunomodulatory, by affecting function and fate of immune cells [4].

The problems related to cell transplantation are primarily associated with the influence of the microenvironment on the properties of cells in a tissue [5]. MSCs have their own set of receptors for growth factors and cytokines that mediate the influence of external factors on intracellular signaling processes and, ultimately, on the function, behavior, and fate of cells.

Autophagy plays an important role in mechanisms that ensure the vital activity of all cell types, supporting their homeostasis, differentiation and various types of activity. It has predominantly protective role defending cells from harmful influences. Autophagy is activated by stressful conditions (starvation, inflammation), cell differentiation, senescence, aging, and many other conditions. In addition, autophagy occurs even in quiescent cells having some functions in resting state. In total, autophagy could provide a promising approach for improving MSC state in application for purposes of regenerative medicine.

## 2. Mesenchymal Stem Cells (MSC)

Mesenchymal stem cells (MSC) are pluripotent cell population, which can be found in almost all tissues. MSCs can be characterized by the presence of a number of specific features that distinguish them and allow this type of cell to actively participate in the regeneration processes at various stages.

MSC were isolated from almost all tissues and organs: bone marrow, adipose tissue, liver, kidneys, muscles, dermis, myocardium, blood vessels, pancreas, thymus, lungs, brain, intestinal crypt, hair follicles, placenta, decidual shell, umbilical cord, and cord blood, from amniotic fluid [6,7]. The standard protocol of MSC isolation includes obtaining the stromal–vascular fraction with subsequent seeding the cell by adhesion to plastic.

As a common feature, MSC isolated from different sources should express CD105, CD73, CD90 (some authors add CD13, CD29, CD44, and CD10) [8,9]. A number of markers (STRO-1, SSEA-4, and CD146) are used to isolate MSC populations with more ‘stem’ properties. Some authors mentioned PDGFRβ, NG2, CD106, α-actin, SCA-1 [10] as MSC markers.

Currently, it is widely recognized that in the MSC population just part of cells satisfies all criteria for mesenchymal stem cells, while other cells are more ‘mature’ (differentiated) or vice versa, similar to embryonic stem cells expressing OCT-4 and SOX2 factors [7]. A comparative expression patterns of MSC derived from several sources revealed a difference in stemness marker genes (SOX2, sex determining region Y-box 2; OCT4, octamer-binding transcription factor 4; KLF4, Kruppel-like factor 4; MYC, NANOG, LIN28, REX1, INHBA), with predominant expression in BM-derived- and adipose tissue MSC [11].

General issue property of MSC regardless of origin, is a capability to differentiate to adipogenic, osteogenic, chondrogenic directions and, concerning to some authors, along myogenic and neurogenic pathways [12]. In addition to the ability to differentiate into osteoblasts, adipocytes, and chondrocytes in vitro, MSCs can be transformed into bone cells and cartilage after ectopic implantation in vivo. In animal models with genetic disorders of bone tissue, it was confirmed that MSC contribute to the regeneration of the bone [13]. Many studies report the ability of MSC to differentiate in vitro or in vivo in several other types of mesodermal cells (myoblasts, endotheliocytes, pericytes, fibroblasts, smooth muscle cells, cardiomyocytes, macrophages) and non-mesodermal origin (hepatocytes, Langerhans islets cells, astrocytes, oligodendrocytes, Schwann cells, neuron-like cells) [10]. The differentiation potential of these cells, as well as a capability to proliferate, makes it possible to use them as an instrument for tissue engineering [14].

The ability of MSC to such multipotent differentiation is not generally recognized. Such problems arise due to the lack of globally standardized methods for the isolation, culture and characterization of MSCs, as well as due to the large variety of methods for determining the terminal-differentiated, functionally mature cell state. The statements of some authors about the differentiation of MSC in vivo to other types of cells are also controversial, since it was shown that bone marrow MSCs are embedded after transplantation in tissue rather through fusion with endogenic cells than through differentiation into mature tissue cells. It really remains unclear to what extent MSCs are actually multipotent [10,15].

Differentiation potential is not the only factor determining MSC participation in regenerative processes. In addition to differentiation, the main intended mechanisms by which MSCs can affect the recipient’s body include immunomodulation, secretion of repairing factors (trophic or paracrine effect), the possible transfer of mitochondria or vesicles containing mRNA, microRNA, and proteins [16,17,18,19,20,21,22,23,24].

MSC express and secrete a wide range of factors governing the immune response, angiogenesis, cell proliferation, migration, invasion, survival and some other processes that promote tissue repair [23,24]. Table 1 illustrates some markers characterizing MSC differentiation, and responsible intracellular mechanisms, as well as the secreted components involved in tissue repair, immunomodulation, and anti-inflammation.

In addition, MSCs have a pericyte-like phenotype and functions that play a crucial role in maturation of blood vessels [29]. It makes them very useful tool for improving tissue repair, tissue engineering and some other applications in regenerative medicine. Mesenchymal stem cells are actively used for treatment of cardiovascular disease [30], metabolic disturbances [31], immune disorders [32], brain injury [33], and many others.

A clinical use of MSC is conditioned by several beneficial properties of these cells, including a possibility to migrate into damaged area, to secrete biologically active substances, and in cases to differentiate.

Improvement of the methods for the clinical application of MSCs can be associated with the preconditioning of cell cultures in a controlled microenvironment [34,35], the creation of structures (“cell sheets”) of high cell density [36,37], the use of extracellular vesicles of MSCs as a source of trophic factors, cytokines, etc. [38,39], and a number of other approaches. Furthermore, the development of genetic methods for controlling MSC differentiation and growth factor secretion also seems to be very promising [40,41,42].

MSC have many applications in medicine, from improvement of pathological state (tissue infarction, degeneration, etc.) to creating a tissue constructs, which can be implant for recovery of tissue function. However, some beneficial, peculiar features of these cells can be improved by the ways, which affect signaling processes providing cell alive, metabolic status, and other vital functions. Currently, the most intriguing and actively developed branch of biological science is autophagy, which plays an active role in almost all aspects of cell life (among other intracellular processes).

## 3. Autophagy: General Aspects

**Figure 1:**
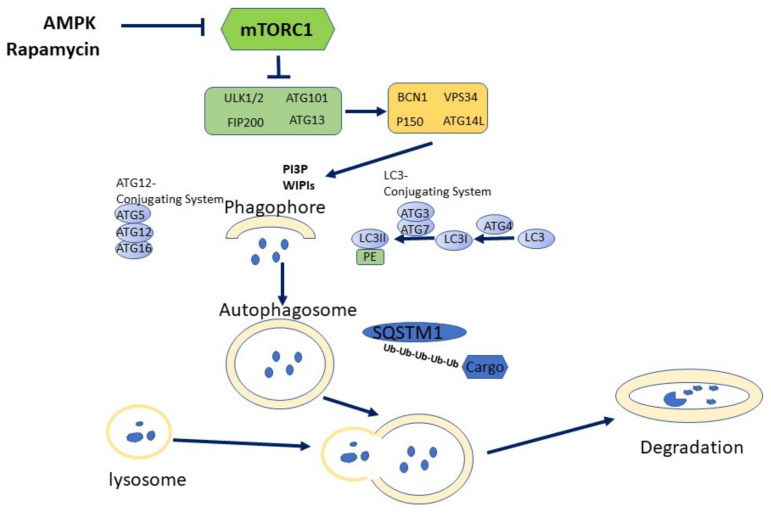
Autophagy signaling. The inhibition of mTORC1 by Rapamycin or AMPK abolishes mTORC1-dependent suppression of ULK1/2 activity and thereby triggers autophagy. Activation of VPS34 results in synthesis of PI3P necessary for phagophore formation. ATG12 and LC3 conjugation systems are involved in elongation and closure of autophagosome membrane. SQSTM1 as well other autophagy receptors, transfer polyubiquitilated cargo into autophagosome. Autophagosome fuses with lysosome following by degradation of content with lysosomal enzymes. Abbreviations used: ULK, Unk-51 autophagy activating kinase; FIP-200, FAK family kinase interacting protein of 200 kDa; BCN1, Beclin 1; VPS34, vacuolar protein sorting 34, class III PI3 kinase; SQSTM1, sequestosome 1; LC3, microtubule-associated proteins 1A/1B light chain 3B.

Autophagy is a conservative intracellular catabolic process by which cells direct their components to lysosomal degradation through formation of autophagosome and subsequently autolysosome. Autophagy encompasses three major types (macroautophagy, microautophagy, chaperon-mediated autophagy) [43], the term ‘autophagy’ usually relates to macroautophagy (see Figure 1).

The process of autophagy begins with the formation of phagophore (the membrane fragment, which originates from the endoplasmic reticulum, Golgi or mitochondrial membrane) which gives rise to autophagosome, - bilayer membrane vesicle with the ‘cargo’ (cell cytosolic components) included in its cavity. The autophagosome is merged with a lysosome, forming an autolysosome, in which (with the participation of proteinases in an acidic medium), the contents are digested [44].

Microautophagy is more simple process, which includes direct invagination of the lysosome membrane and proteolysis of their contents [45]. In chaperone-mediated autophagy target proteins containing a signal KFERQ-like pentapeptide are recognized by the HSC70 cytosolic chaperone, which mediates protein translocation in the lysosomes via LAMP2a receptor [46].

The autophagy is performed by several protein complexes, which are formed mainly by proteins denoted as ATG (‘AutoPhagy-Related Gene’). ULK1/ULK2 complex (UNC-51-like kinases, mammalian homologue of ATG1) initiates phagophore formation and membrane nucleation [47]. In addition to ULK protein kinase, this complex includes factors ATG 13, ATG101 and FIP200/ATG17. ULK kinase phosphorylates and activates BECLIN-1 (mammalian homolog of ATG6), which is as part of a complex including P150/VPS15, ATG14L, and VPS34 proteins, -phosphatidylositol-3-phosphate type III type (PI3K Type III) [48].

Phosphorylation of BECLIN-1 by ULK-complex attracts phosphoinositide-3-kinase VPS34 to the area of a double membrane formation. In turn, VPS34 kinase synthesizes phosphatidylositol-3-phosphates on the membrane as a signal of autophagosome formation, which attracts the ATG protein complex to the membrane [49]. The covalent complex of the ubiquitin-like ATG12 protein with ATG5, which appears in the process of phagophore formation, attaches an ATG16L protein with the participation of WIPI2 factor that binds phosphatidylositol-3 phosphate (Ptdins3p), formed by the VPS34 kinase [50,51].

This complex is involved in lipidation (attachment of phosphatidylethanolamine) microtube-associated light chain protein LC3. LC3 is the main mediator of the elongation of the membrane of autophagosomes, the recognition of the target and the merging of autophagosomes with lysosome [52,53]. Lipidated LC3 protein contributes to the closure of autophagosome and allows it to keep the components with transition proteins in the autophagosome, such as sequestosome-1 (SQSTM-1/p62) [54].

Selective-autophagy relies on the recognition of poly-ubiquitylated targets by specific autophagy receptors, including NBR1, optineurin, and SQSTM1/p62 [55]. The entrance of cytosolic constituents into phagosome and autolysosome is supplied by specific receptors associating with cytosolic components (protein aggregates, misfolded or excessive proteins) via ubiquitination and/or polyubiquitilation.

An example of autophagy receptor, -sequestosome-1, contains domains that mediate its interaction with LC3, components of intracellular signaling and ubiquitylated protein components intended for degradation [56,57]. It can be suggested that such a receptor structure makes it possible to bind selective proteins for autolysosomal degradation, and may serve as a mechanism modulating cellular signaling and more complex processes.

The source of membrane for mammalian autophagosome elongation are endoplasmic reticulum, Golgi complex, mitochondria, or plasma membrane [58,59]. Stimulation of autophagy can proceed via extra- and intracellular stress, starvation, deprivation of growth factors, stress of the endoplasmic reticulum, as well as various types of pathogenic infection. The main regulators of autophagy are mTOR protein kinase complex 1 [60,61], which in an active state suppresses autophagy, and AMP-dependent protein kinase [62] activating this process. The regulatory effect of these factors is mediated by modulation of the activity of the ULK1/ULK2 kinase, which initiates the autophagy process.

Mammalian/mechanistical target of rapamycin (mTOR) exists in the form of two complexes, mTORC1 and mTORC2. mTORC-1 is active in excess of nutrients, and suppresses autophagy by phosphorylation of ULK1. On a contrary, in nutrient deficiency (starvation) mTORC-1 is inactive, and ULK1/2 becomes activated following by autophagy induction.

Adenosine monophosphate-activated protein kinase (AMPK) is a crucial cellular energy sensor (as AMP/ATP ratio), which plays a significant role in the regulation of autophagy. Under nutrient deficiency, AMPK activity is elevated with a related increase of the AMP/ATP ratio, resulting in direct ULK1 activation and autophagy induction [63].

## 4. MSC Differentiation and Autophagy

Mesenchymal stem cells can differentiate into several specific cell lineages under appropriate stimuli [64]. The process of cell differentiation is always very complex and multistage. However, it is possible to highlight its most characteristic markers, like PPARγ (peroxisome proliferator activated receptor) as a marker and master gene of MSC adipogenic differentiation, which proceeds at the expense of osteogenesis [65], and Runx2 (Runt-related transcriptional factor 2) as a necessary component of signaling resulting in osteogenic differentiation, as well as the expression of osterix/SP7 [66] (Table 1).

Autophagy is a necessary factor of adipogenic differentiation. MSC knockout on component(s) of autophagy signaling (ATG7, ATG5) results in downregulation of triglyceride accumulation, diminished expression of C/EBPα, PPARγ, aP2, Glut4, and other proteins characterizing adipocyte maturation. These changes occurred along with increasing expression of markers of mitochondria biogenesis (UCP1, PGC-1α, cytochrome oxidase, cytochrome c), in gonadal white adipose tissue [67].

**Figure 2:**
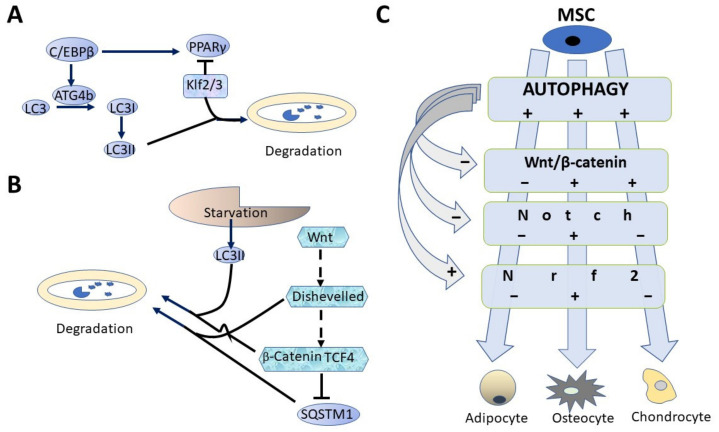
Relationship between autophagy and intracellular signaling participating in MSC differentiation. (**A**) adipogenesis transcription factor C/EBPβ activates proteinase ATG4b, which induces LC3 maturation. LC3II binds factors Klf 2/3 and transfers them into autolysosome, thereby eliminating PPARγ suppression by Klfs [69]. (**B**) LC3II and SQSTM1 perform autolysosomal degradation of Disheveled (Dvl) and β-catenin. This effect antagonizes SQSTM1 suppression by Wnt/β-catenin signaling [71,72]. (**C**) autophagy is a necessary factor of MSC differentiation; Wnt and Notch weaken adipogenesis and reinforce osteogenesis. The existence of selective relationships between autophagy and cell signaling creates an opportunity to regulate efficiency of MSC differentiation as a factor of tissue repair.

CCAAT enhancer binding protein β (C/EBPβ), a factor important for adipogenesis, is also required for activation of autophagy, as shown on 3T3 L1 cells. ATG4b, cysteine proteinase necessary for LC3 maturation, is a target gene of C/EBPβ. Its expression prompts degradation of Klf2 and Klf3, two inhibitory factors of adipogenesis [68], via ubiquitination in SQSTM1/p62-dependent manner (Figure 2A) [69].

At the same time, if autophagy activation can be achieved by pharmacological agents, such as gamma-tocotrienol or fluoxetine, then suppression of adipogenesis at the early stage can be observed. However, it is not clear if autophagy and adipogenesis are interrelated or simply parallel processes [70]. These data demonstrate the occurrence of delicate balance between basal level and induced autophagy as a regulatory power of cellular fate.

One can assumed that autophagy is a necessary condition for a change in the cellular phenotype during differentiation, since this process requires a balance between degradation and synthesis of new cellular components. At the same time, the activation of ‘excessive’ autophagy can affect some elements of differentiation, especially in the early stages, thereby preventing it.

It is interesting to note a relationship between Wnt/β-catenin signaling pathway, a negative regulator of adipogenic differentiation, and autophagy. The Wnt/β-catenin pathway is a negative regulator of both basal and stress-induced autophagy [71]. β-Catenin suppresses autophagosome formation and directly represses SQSTM1/p62 with participation of Wnt signaling factor TCF4. Nutrient deficiency leads to selective β-catenin degradation via β-catenin–LC3 complex formation, attenuating thereby β-catenin/TCF-driven transcription (Figure 2B). The β-catenin–LC3 complex is formed via W/YXXI/L motif and LC3-interacting region (LIR) in β-catenin. Thus, Wnt/β-catenin represses autophagy and p62 expression, while β-catenin itself is targeted for autophagic clearance in autolysosomes upon autophagy induction [71].

The activity of autophagy as a negative regulator of Wnt signaling can be also mediated by promoting degradation of Disheveled (Dvl), which is another participant of Wnt signal transduction. This process is mediated via Dvl2 ubiquitylation by E3 ubiquitin ligase, Von Hippel–Lindau protein. This is critical for binding of Dvl2 to SQSTM1/p62, which in turn governs its transport into autolysosome [72].

Wnt signaling plays indispensable role in adipocyte biology by regulating adipose tissue dynamics and metabolism [73]. Any disturbances of this pathway, either acquired or hereditary, can result in somatic and metabolic diseases, the type 2 diabetes as well [74]. To search possible cross-roads between Wnt and autophagy should help to follow general mechanisms supplying MSC homeostasis and differentiation modes.

Adipose tissue is insulin-dependent, as well as skeletal muscle and liver. It is suggested that the disturbance of its physiological state in obesity gives rise to systemic insulin resistance and type 2 diabetes mellitus (T2DM) [75].

It should be noted that autophagy disorder could decrease an efficacy of lipid mobilization and result in adipocyte hypertrophy, contributing to development of latent inflammation in adipose tissue associated with insulin resistance and T2DM [76].

The assessment of autophagy level in patients with obesity and in animal models have shown that obesity is accompanied by increase of ATGs that correlates with adipose tissue inflammation [77]. These data could suggest that inflammation induced by hypoxia of adipose tissue in its latent state is a detrimental factor of development and metabolism of adipose tissue.

Osteogenic differentiation of MSC also needs autophagy to perform. Vidoni et al. [78] have shown that gingival MSC osteogenesis proceeds along with autophagy evaluated by autophagosome/lysosome fusion. At the same time, presence of specific and potent autophagy inhibitor-1 (spautin-1) in culture medium during osteogenesis greatly reduced osteogenic marker expression, as well as autophagy suppression.

Bone marrow MSCs (BMMSC) of osteoporotic patients which were characterized by low osteogenic capability with senescent phenotype (galactosidase staining) and low LC3II/LC3I ratio), demonstrated improved osteogenesis upon autophagy stimulation by rapamycin. These changes were reversed by autophagy inhibition with 3-MA [79].

Similar results obtained in mice. BMMSC of young animals were more committed to osteogenic differentiation, demonstrated higher proliferative activity, higher level of autophagy than that of older mice. Remarkably, autophagy activation by rapamycin in older animals, as well as its inhibition in youngers ameliorated this difference [80].

Activation of Wnt/β-catenin signaling in MSC stimulates osteoblastogenesis and inhibits adipogenesis by modulating the relative levels of cell type specific transcription factors [81]. Chondrogenic differentiation of MSC is one more point of these cells application for tissue regeneration having some more advantage in comparison with autologous chondrocytes [82].

Autophagy greatly contributes to chondrogenic differentiation. The ablation of autophagy gene Atg7 impaired MSC transition to chondrocytes. Atg7-deficient chondrocytes accumulated large numbers of glycogen granules, were growth-retarded, and died without signs of endoplasmic reticulum stress. Interestingly, in addition to other compensatory mechanisms, autophagy participates in glycogenolysis to supply glucose in avascular growth plates [83].

Synovium-derived mesenchymal stem cells (SMSCs) exhibiting superior chondrogenesis represent promising cells for cartilage tissue engineering. However, inflammatory cytokines such as IL-1β hamper chondrogenic differentiation by decrease of SOX9, aggrecan, and collagen II expression. In parallel, IL-1β upregulated the expression of mTOR expression, decreased LC3-II/LC3-I ratio and autophagosome formation. Rapamycin potentiated MSC chondrogenic differentiation, in addition to promotion of autophagy. Effect of rapamycin depended on Wnt signaling because it was abolished by GSK3β inhibitor [84].

Wnt/beta-catenin signaling regulates chondrogenic and adipogenic differentiation of pericytes. Inducing Wnt/beta-catenin signaling triggers the chondrogenic differentiation and at the same time weakens differentiation into adipocytes [85].

In summary, one can assume that the relationship exists between autophagy and the signal transduction mechanisms that mediate MSC differentiation. This connection is illustrated in Figure 2C as positive (+) or negative (−) action of autophagy on signal transduction, personified by Wnt/β-catenin, Notch and Nrf2 (see below) resulting ultimately in modulating effects on cell differentiation.

## 5. Immunomodulatory Activity of MSC

Immunomodulation is a promising field for application of MSC in treatment of sepsis, use in transplant medicine, autoimmune disease. Immunomodulatory action of MSC encompasses effects on monocytes and macrophages, dendritic cells, lymphocytes [86].

In addition, immunomodulatory function of MSC could play a significant role in tissue repair, since immune cells (macrophages) actively participate in reparative and regenerative process [87].

Immunomodulation can be characterized as a paracrine action of MSC on immune cells inducing an acquisition anti-inflammatory features by immune cells [88]. At the same time, a result of cross-talk between mesenchymal stem cells and immune cells can depend on MSC state [89].

In this regard, the great interest represent studies performed by group of A. Betancourt [35,36]. These authors have shown that MSC preconditioning with proinflammatory factors/cytokines (LPS, TNFalpha, INFgamma) acquire a proinflammatory phenotype, i.e., a capability to express and secrete proinflammatory factors. On another hand, ani-inflammatory preconditioning results in acquiring the anti-inflammatory properties of MSC. This phenomenon is very similar to polarization of inflammatory cells, macrophages, and T cells, into proinflammatory (M1) and anti-inflammatory (M2) phenotype. Among T cell population different types, Th1, Treg, and Th2 can be distinguished as a result of polarization (‘preconditioning’) into cells with different inflammatory status. One can await that the modulation of autophagy, reflecting on the features of MSC can change their immunomodulatory capability.

Indeed, MSC pretreatment with rapamycin (which enhances autophagy flow by inhibiting mTORC1) improves immunosuppression by inducing cyclooxygenase-2 and prostaglandin E2 upregulation [90]. This mechanism determines significant anti-inflammatory therapeutic effect in animal model [91]. In another study, TGFbeta, as well as indolyl 2,3-dioxygenase and interleukin 10 increased on the treatment of MSC by rapamycin and determined immunosuppressive effect on CD4+ T cells [92].

It should be noted that stimulation of MSC autophagy by rapamycin affects immunomodulatory action, by changing T cell migration capability as well as inducing their differentiation [93]. Autophagy inhibitor 3-methyl adenine exerted an opposite action abolishing the effects of rapamycin.

## 6. Some Signal Processes Involving Autophagy

### 6.1. Notch

A significant aspect of cell fate determining is cell–cell interaction in multicellular development. Notch signaling pathway is one of fundamental, conservative cellular interaction mechanisms that regulate a number of cellular processes including cell functions and differentiation [94].

Notch signaling system in cell is represented by 4 transmembrane Notch receptors (Notch-1, -2, -3, -4), 5 canonical (DLL1, DLL3, DLL4, Jagged1, Jagged2) and 2 non-canonical (DLK1 and DLK2) ligands. Receptor-ligand interaction renders sequential receptor cleavage in signal-accepting cell by metalloproteinase ADAM and by γ-secretase complex resulting in release of Notch Intracellular Domain (NICD) that translocates into nucleus where it forms transcriptional complex with SCL transcriptional factor modulating transcription of target genes such as HES family. Notch signal transduction can be efficiently inhibited by pharmacological γ-secretase inhibitors, among them, DAPT (N-[N-(3,5-difluorophenacetyl-L-alanyl)]-S-phenylglycine t-butylester) is a frequently used for study of Notch effect on cellular and organismal level.

Song et al. [95] demonstrated that inhibition of Notch signaling in bone marrow MSC by DAPT promoted adipogenic differentiation (as estimated by Oil Red staining and PPARγ expression). This potentiation proceeded along with activation of autophagy caused by inhibition of PI3K/mTOR signaling. Autophagy inhibitors, chloroquine, and 3-methyl adenine, both suppressed MSC adipogenesis.

Evidently, Notch activation should be inhibitory for MSC adipogenic differentiation and autophagy. At the same time, lentiviral transduction of Notch signaling components, NICD or Jagged1, into adipose tissue MSC enforced osteogenic differentiation in a dose-dependent manner [96].

The relationships between Notch and MSC differentiation can be not so simple. For instance, DLK1/Pref1, a component of Notch, suppresses adipo- and osteo-genesis [97] by preventing degradation of SOX9, along with promotion of chondrogenic induction [98]. Thus, by preserving the expression of SOX9, Pref1 is able to keep MSC in undifferentiated state.

According to data of Song et al. [95], Notch signaling is inhibitory for adipose differentiation of MSC. During adipose differentiation Notch-signaling is inhibited in autophagy-dependent manner, and autophagy inhibition, along with suppression of adipogenesis, induces Notch activation.

### 6.2. Hypoxia Inducible Factor (HIF-1α)

HIF-1α if a member of transcription factors which are sensitive to oxygen concentration in media. At normal oxygen concentration, HIF-1α is modified by prolylhydroxylase with subsequent proteosomal degradation mediated by von Hippel–Lindau E3 ubiquitin ligase, which is inhibited by low oxygen, thus preventing HIF-1α cleavage.

Lentiviral hyperexpression of HIF-1α in bone marrow MSC increased cell viability in oxygen-glucose deprivation condition. HIF-1α contributes to MSC survival and efficacy of MSC application for treatment (by cell transplantation) cerebral infarction in the animal model of cerebral artery occlusion. MSC with HIF-1α hyperexpression promoted reduction of brain infarct volume, improved neurobehavioral outcome and, additionally, inhibition of proinflammatory cytokine secretion while promotion of neurotrophin release. AMPK was activated while mTOR was inactivated by HIF-1α overexpression suggesting autophagy participation in these effects [99]. The data were supported by another group [100] that have shown that hypoxic preconditioning is mediated by autophagy via BNIP3–Beclin1 pathway.

Liu et al. [101] have shown that HIF-1α and autophagy are both responsible for increased motility and invasiveness of human endometrial stromal cells (HESCs), induced by hypoxia. In addition, hypoxia HIF-1α-dependently changes differential potential of MSCs, facilitating osteogenic differentiation and hampering adipogenesis [102].

One should note that coupling between hypoxia/HIF-1α and autophagy is not unique for MSC, and could be met in other cell types. As an example, a study of Belibi et al. [103] have shown colocalization of HIF-1α and autophagy-related structures (autophagosomes, mitophagy, and autolysosomes revealed by electron microscopy) in the tubular cells lining the cysts in the model of polycystic kidney disease.

### 6.3. Nrf2/Keap1 Axis

Oxidative stress, in particular reactive oxygen species, have a significant contribution to cellular physiology, by activating many processes including cell differentiation. Usually, oxidative stress is transient, and phase of its activation is changed for oxidative quenching due to activity of antioxidant mechanisms. A possibility to regulate cellular expression of antioxidant genes is represented by nuclear factor erythroid 2-related factor 2 (Nrf2), a transcription factor acting as a potent anti-oxidant regulator.

The transcriptional activity of (Nrf2) is regulated by cytosolic-nuclear transition, and by ubiquitination mediated by Keap1 (Kelch-like ECH-associated protein 1). In basal conditions, Nrf2 bound to keap1 is subjected to proteosome degradation [104].

In stressful conditions, Nrf2 is released from binding to keap1, migrates to the nucleus and binds to anti-oxidant response element (ARE) [105]. Nrf2 activates ARE-dependent genes in that number glutathione (GSH), heme oxygenase 1 (HO1), NAD(P)H quinone oxidoreductase-1 (NQO1), γ-glutamyl cysteine ligase catalytic subunit (GCLC), and many others [106,107].

Autophagy is activated along with other processes by oxidative stress via several mechanisms. ROS have several application points to activate autophagy, mainly via pathways which usually mediate autophagy modulation in conditions of starvation (mTORC1, AMPK), RedOx fluctuations, inflammatory conditions [108,109]. In turn, autophagy mobilizes mechanisms preventing sustain activation of oxidation. An example of such stimulation is Nrf2 mobilization, mediated by sequestosome, SQSTM1/p62.

Sequestosome-1 is a multidomain protein, containing among others KIR (keap1-interacting region) domain, which interact with keap1 and brings it out from connection with NRF2, resulting in Nrf2 activation [110,111].

Moreover, p62 not only binds keap1, but also removes it by autophagy mechanism, via ubiquitination with subsequent autolysosomal degradation. This is an example demonstrating a possibility of autophagy to influence intracellular signal transduction and selective protein expression [55,112].

Another possibility to activate Nrf2 (and to induce antioxidative effect) consists in keap1 modification (alkylation) by itaconate, a tricarbonic acid cycle metabolite [113,114].

Nrf2 prevents MSC differentiation into osteocytes induced by autophagy activation [115]. Thus, Nrf2 mobilization can serve as a factor to regulate cell differentiation.

Nrf2 mobilization by autophagy (via keap1 elimination) can increase antioxidant potential of cell. At the same time, cell differentiation could be potentiated by stress, which in turn can be induced by autophagy. ROSs activate autophagy that facilitate cellular adaptation and diminishes oxidative damage by degrading and recycling intracellular damaged macromolecules and dysfunctional organelles [102].

## 7. Conclusions and Perspectives

Autophagy plays a multiform role in cellular life. By degrading cellular components, autophagy replenishes energy sources in deficiency of nutrients; by removing damaged cellular components and organelles, autophagy promotes cell survival, and also affects energy metabolism, mitigating the effects of various types of stress.

The function of autophagy is closely associated with other cellular systems that provide vital activity and the performance of cellular function. Almost all the basic functions of the cell are somehow associated with autophagy. This circumstance is directly related to the processes of cell differentiation and other functions of MSCs.

It is paradoxical that autophagy is necessary for all types of MSC differentiation; however, adipogenic, osteogenic, and chondrogenic differentiations are fundamentally different from each other. Apparently, the resolution of this paradox is the possibility of the impact of autophagy to additional signaling processes involved in cell differentiation.

In particular, the effect of autophagy on Wnt/β-catenin, Notch, Nrf2/keap1 signaling, as well as other types of signal transduction involved in cell differentiation and cell function, explains the specificity of autophagy involvement in differentiation in various directions. Thus, autophagy is not a trigger but rather a modulator of cellular processes.

It is the modulating role of autophagy that manifests itself in those cases, when, by degrading one of the components (b-catenin, keap1), autophagy affects the signaling process (Figure 1). Thus, the role of selective autophagy may consist not only in the degradation of selective organelles, but also of individual components of the cell.

It is of certain interest to study the interactions of autophagy with cellular signaling mediating various types of MSCs functional activity, with the aim of their subsequent use as targets for improving proliferative, differentiating activities and paracrine effects on other types of cells.

In this regard, it deserves a lot of attention a functionality of autophagy receptors (SQSTM1, OPTN, and others), as well as principles governing a selectivity of function of proteosomal and autolysosomal degradation of cell components.

## Figures and Tables

**Table 1:** Factors having impact on MSC differentiation and production of MSC-derived repairing and immunomodulatory effectors.

MSC Activity	Markers	Responsible Mechanisms	Modulators	Reference
MSC differentiation				
Adipogenic	Glut4, Perilipin-2, PGC1α, Pref1, UCP-1, aP2	CEBPα, PPARγ	Klf2/Klf3 Pref1	[25]
Osteogenic	ALPP, SPARC, collagen I	RUNX2, Osterix	LY3023414	[26]
Chondrogenic	Annexin A6, CD44, CD151, ITM2A, collagen II/IV	FAM20B, FoxC1, Fox C2/SOX9	SOX9, Il-1β	[27]
MSC secreted factors				
Tissue repair/angiogenesis	VEGF, HGF, EGF, TNFα, MIP-1, TIMPs, IL6, IL8	Pro-/anti-inflammatory signaling, MAPK kinases	Cell signaling inhibitors	[23,25]
Immunomodulation	IDO, TGFβ, HGF, PGE2	Pro-/anti-inflammatory signaling, MAPK kinases	Cell signaling inhibitors	[28]

Abbreviations used: ALPP, Alkaline Phosphatase; SPARC, secreted protein acidic and rich in cysteine; CEBP, CCAAT/enhancer-binding protein; FAM20B, enzyme phosphorylating the xylose residue in the glycosaminoglycan-protein linkage region; Glut-4, Glucose transporter type 4; ITM2A, Integral membrane protein 2A; KLF, Kruppel like factor; PGC1α, Peroxisome proliferator-activated receptor gamma coactivator 1-alpha; PPAR, peroxisome proliferator activating receptor; Pref1/DLK1, preadipocyte factor 1/delta-like 1; RUNX2, Runt-related transcription factor 2; SPARC, Secreted protein acidic and rich in cysteine; UCP-1, Uncoupling protein 1.

## Data Availability

Data sharing not applicable to this article as no datasets were generated or analysed during the current study.
